# The role of subcortical grey matter structures in neuropsychiatric symptoms from cognitively healthy tot mild cognitive impairment: a population‐based study

**DOI:** 10.1002/alz.093875

**Published:** 2025-01-09

**Authors:** Ilse vom Hofe, Annemarie I. Luik, Meike W. Vernooij, M. Arfan Ikram, Frank J. Wolters

**Affiliations:** ^1^ Erasmus University Medical Center, Rotterdam Netherlands; ^2^ Erasmus University Medical Center, Rotterdam, Zuid‐Holland Netherlands

## Abstract

**Background:**

Neuropsychiatric symptoms are common in the prodromal phase of dementia, and may be related to altered structure and function of subcortical grey matter involved in emotion regulation. We aimed to determine the association between subcortical grey matter volume and occurrence of neuropsychiatric symptoms in a population‐based study.

**Method:**

Between 2009‐2015 dementia‐free participants from the population‐based Rotterdam Study underwent 1.5T brain MRI. Volumes of the accumbens, amygdala, caudate, hippocampus, pallidum, putamen and thalamus were determined using FreeSurfer 6.1. Symptoms of depression and anxiety were assessed using the Center for Epidemiologic Studies Depression (CES‐D) scale and the anxiety subscale of the Hospital Anxiety and Depression scale (HADS‐A). We determined cross‐sectional associations between subcortical volumes (per 1 standard deviation [SD] decrease) and continuous CES‐D and HADS‐A scores using linear regression, and with clinically significant symptoms (CES‐D=16;HADS‐A=8) using logistic regression. Models were adjusted for demographics, lifestyle factors, comorbidities and co‐medication use. We repeated analyses stratifying on cognitive status (healthy/subjective complaints/MCI).

**Result:**

Of 3452 participants (mean age 69.3 years, 55.2% women), 284 (8.2%) scored positive for clinical depressive symptoms, and 270 (7.8%) for clinical anxiety symptoms. Smaller hippocampal volume was associated with higher odds of clinical depressive symptoms (odds ratio [95%CI]: 1.27[1.05‐1.52], p=0.01). In contrast, smaller volumes of the caudate were associated with less depressive symptoms (mean difference in log‐transformed score [95%CI] for caudate: ‐0.06[‐0.10;‐0.02], p<0.01, and pallidum: ‐0.04[‐0.08;0.00], p=0.05). Smaller volumes of the caudate and pallidum were also associated with less anxiety (caudate: ‐0.04[‐0.07;‐0.01], p<0.01, pallidum: ‐0.05[‐0.09;‐0.02], p<0.01), accompanied for the caudate by a lower odds of clinical anxiety symptoms (OR[95%CI]: 0.84[0.72;0.98], p=0.03). No associations were observed with the accumbens, amygdala, putamen, or thalamus. Associations were observed in cognitively healthy participants, as well as in those with subjective cognitive complaints or MCI.

**Conclusion:**

In community‐dwelling older adults, volumes of the hippocampus, caudate, and pallidum are associated with neuropsychiatric symptoms, which do not differ by neurodegenerative disease stage. These findings support a complex role of subcortical volumes in the occurrence of neuropsychiatric symptoms in the older population.

